# The contradictory role of androgens in cutaneous and major burn wound healing

**DOI:** 10.1093/burnst/tkaa046

**Published:** 2021-04-20

**Authors:** Huaikai Shi, Kenny Cheer, Ulla Simanainen, Brian Lesmana, Duncan Ma, Jonathan J Hew, Roxanne J Parungao, Zhe Li, Mark S Cooper, David J Handelsman, Peter K Maitz, Yiwei Wang

**Affiliations:** 1 Burns Research Group, ANZAC Research Institute, University of Sydney, Concord Hospital, Gate, 3 Hospital road, Concord, NSW 2139, Australia; 2 Andrology, ANZAC Research Institute, University of Sydney, Concord Hospital, Gate, 3 Hospital road, Concord, NSW 2139, Australia; 3 Adrenal Steroid Laboratory, ANZAC Research Institute, University of Sydney, Concord Hospital, Gate, 3 Hospital road, Concord, NSW 2139, Australia; 4 Burns and Reconstructive Surgery Unit, Concord Hospital, Gate, 3 Hospital road, Concord, NSW 2139, Australia

**Keywords:** Wound healing, Androgen, Mouse model, Oxandrolone, Cutaneous injury, Major burn injury

## Abstract

Wound healing is a complex process involving four overlapping phases: haemostasis, inflammation, cell recruitment and matrix remodeling. In mouse models, surgical, pharmacological and genetic approaches targeting androgen actions in skin have shown that androgens increase interleukin-6 and tumor necrosis factor-α production and reduce wound re-epithelization and matrix deposition, retarding cutaneous wound healing. Similarly, clinical studies have shown that cutaneous wound healing is slower in men compared to women. However, in major burn injury, which triggers not only local wound-healing processes but also systemic hypermetabolism, the role of androgens is poorly understood. Recent studies have claimed that a synthetic androgen, oxandrolone, increases protein synthesis, improves lean body mass and shortens length of hospital stay. However, the possible mechanisms by which oxandrolone regulates major burn injury have not been reported. In this review, we summarize the current findings on the roles of androgens in cutaneous and major burn wound healing, as well as androgens as a potential therapeutic treatment option for patients with major burn injuries.

Highlights

- In this article, we review the current mouse models used in investigating the actions of androgens in wound healing.
- The role of androgens in cutaneous and burn injury wound-healing processes is discussed.
- The clinical evidence for the use of oxandrolone in treating major burn injury is reviewed.
- Finally, we highlight the potential of dihydrotestosterone as a therapeutic approach in burn wound healing.

## Background

Cutaneous injuries and small and major burn injuries trigger a wound-healing process which consists of several highly integrated and overlapping phases, including inflammation, cell recruitment, matrix deposition, epithelization and tissue remodeling. In additional to local wound repair, large or major burns also stimulate a systemic hypermetabolic catabolic condition and pathophysiological stress response. If not corrected in time, the hypermetabolic response results in a catabolic state, characterized by weight reduction, a negative nitrogen balance, loss of lean body mass, impaired wound healing and sepsis [1]. These syndromes are associated with delayed recovery, prolonged hospital admission and increased morbidity and mortality.

In men, testosterone is the predominant circulating androgen, which, in target cells, can be rapidly, irreversibly and almost completely converted to the more potent androgen dihydrotestosterone (DHT) by 5α-reductases, with type 1 and type 2 being highly expressed in skin [2, 3]. Androgens are known to exacerbate inflammation and the tissue remodeling phases in cutaneous (non-burn) wound healing. In contrast to the retarding effect of androgens in cutaneous wound healing, a few clinical studies reported using the androgen analog oxandrolone in the treatment of major burn injuries in children, increased protein synthesis, improved lean body mass and shortened length of hospital stay. In the present article, we discuss the experimental models used to study androgenic effects in wound healing, if and how the actions of androgens modify the wound-healing process under a pathophysiological stress response and the role of androgens in treating major burn injuries.

## Review

### Androgens and androgen action

Androgens are a group of 19-carbon steroid hormones produced de novo mainly in testes and, to a much lesser extent, other steroidogenic tissues (ovaries, adrenals and placenta) by the conversion of pro-androgen precursors in peripheral tissues including the liver, skin, adipose tissue, breast and prostate [4, 5]. Androgens have both virilizing and anabolic effects on their target tissues, mediated by binding to and activating the androgen receptor (AR) [5–7].

#### Testosterone and dihydrotestosterone

**Figure 1.**
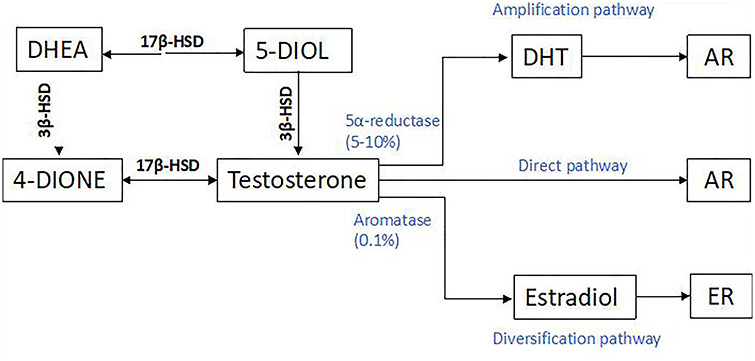
The pathway for synthesis of androgens from precursors DHEA [106]. *DHEA* dehydroepiandrosterone, *DHT* dihydrotestosterone, *3β-HSD* 3β-hydroxysteroid dehydrogenase deficiency, *AR* androgen receptor, *ER* estrogen receptor

The major circulating androgen in male mammals is testosterone, which is secreted by testicular Leydig cells. After testicular secretion, a small proportion of circulating testosterone (~5–10%) is irreversibly converted to the more potent androgen DHT by 5α-reductase enzymes [5, 6]. Testosterone also forms the obligate precursor for the synthesis of estradiol by the enzyme aromatase, thereby diversifying the effects of testosterone due to its activation of estrogen receptors (Figure 1). Two isoforms of 5α-reductases, namely type 1 and type 2 5α-reductase, convert testosterone to DHT, with type 1 predominantly being expressed in the skin [2, 3] (Figure 1).

**Table 1:** Comparison of human and mouse skin histology [11]

**Trait**	**Human**	**Mouse**
Hair coat	Sparse	Dense
Epidermis	Thick	Thin
Dermis	Thick	Thin
Panniculus carnosus	None	Present
Skin architecture	Firmly attached	Loose
Wound-healing mechanism	Re-epithelialization	Contraction

**Figure 2.**
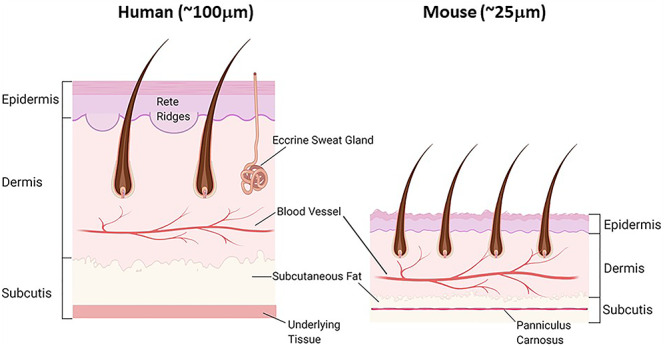
Comparison of human and murine skin structure

#### Synthetic androgens

Since the discovery of testosterone as the principal mammalian testicular-derived androgen in the mid-1930s, thousands of synthetic steroidal androgens have been produced, and in the late 1990s the first non-steroidal androgen was synthesized. Testosterone is used exclusively for replacement therapy in men having pathological hypogonadism or disorders of the hypothalamus, pituitary or testes, which reduce endogenous testosterone production [5, 8–11]. By contrast, synthetic androgens are used for numerous non-reproductive disorders to exploit their effects on muscle size and strength and haemoglobin function, as well as other androgenic effects. However, the clinical applications of synthetic androgens are limited by their virilizing effects for women and children. As testosterone has negligible oral bioactivity due to rapid degradation in the gut and liver, many synthetic androgens have been developed to be orally active—a pharmacological feature which is almost exclusively due to the introduction of a 17 α-alkyl substituent into the molecule. However, oral 17 α-alkylated steroid androgens (e.g. methyltestosterone, fluoxymesterone, oxymetholone and oxandrolone) are hepatotoxic due to the class-specific effects of the 17 α-alkyl group and are therefore not suitable for long-term androgen treatment [5]. Oxandrolone is an orally active synthetic 17-α alkylated androgen with greater potency than testosterone [12, 13]. In addition to being a potent androgen, oxandrolone has additional effects antagonizing glucocorticoid receptor effects [13]. In the absence of any safe oral androgens in the USA, oxandrolone has been used in major burn injury and various other paediatric growth and clinical situations as a synthetic androgen, despite the hepatotoxicity risk, whereas in other Western countries oral testosterone products are available and free from hepatotoxicity. In any situation where oxandrolone has proved clinically effective, its use could be replaced by safer non-hepatotoxic androgens, such as testosterone or DHT.

### Experimental murine models to study androgenic effects on cutaneous wound healing

Much of our current understanding of the role of androgens in wound healing is derived from mouse studies, despite the differences in skin structure, wound healing method and postburn metabolism in mice and humans (Table 1). Wound healing in both mice and humans occurs by different primary mechanisms due to the global presence of the *panniculus carnosus* in murine skin (Figure 2). Contraction of the *panniculus carnosus* assists greatly in reducing wound size in mice, whereas in humans, who lack a *panniculus carnosus*, wound healing occurs primarily by re-epithelialization and granulation [14, 15]. All four wound-healing phases are apparent in both human and murine skin, however, the regulation of the wound-healing process by the immune system and inflammatory response is somewhat different. Specialized immune cells known as λd dendritic cells are found only in mice [16]. During the inflammatory phase, neutrophils are the primary cells recruited to the wound. In humans, neutrophils express antimicrobial defensins that are not expressed by neutrophils in mice [17]. Despite these differences, mice still mirror many aspects of human skin structure and wound-healing processes.

Mouse models of wound healing remain key to understanding wound-healing pathophysiology and the development of new therapies. The homogeneity between individuals, low cost, rapid breeding, ease of handling, greater feasibility to study large sample sizes, shorter wound-healing time and the availability of a wide and versatile array of genetically modified mouse lines means that such models provide a wealth of opportunity and source of insight into wound healing. Furthermore, with appropriate recognition of special differences or limitations, these models are indispensable for future research.

Experimental mouse models have been established to study the androgenic effects on cutaneous wound healing, including orchidectomy [18–21], pharmacological approaches using various AR agonists and antagonists [18, 22], and genetically modified gene-knockout models targeting androgen and/or estrogen synthesis or action [23, 24].

#### Orchidectomy in mice

Orchidectomy removes ~95% of all circulating androgens, but a small residue continues to be produced by the adrenal glands and by peripheral interconversion of pro-androgen precursors. In conjunction with orchidectomy, androgen treatment can be used to investigate specific androgenic effects [25–27]. However, such effects are dose-dependent and vary according to the type of androgen (i.e. whether it is aromatizable or not); additionally, such treatment may exert non-physiological effects at high does. Steroid receptor antagonists, including AR-antagonists (flutamide, bicalutamide, nilutamide, enzalutamide) and steroidogenic enzyme inhibitors, such as 5α-reductase inhibitors (finasteride, dutasteride), have been used to study the general and specific effects of androgen in wound healing [18, 22]. The use of androgen antagonists in an intact mouse will produce countering reflex effects due to negative feedback, leading to increased endogenous testosterone, which may compromise interpretation. While both surgical and pharmacological approaches in mouse models provide useful, albeit limited, insights, our understanding of androgenic action has been boosted by the introduction of genetic methods involving the global or tissue-selective knockout of AR function.

#### Global knockout mouse models

**Figure 3.**
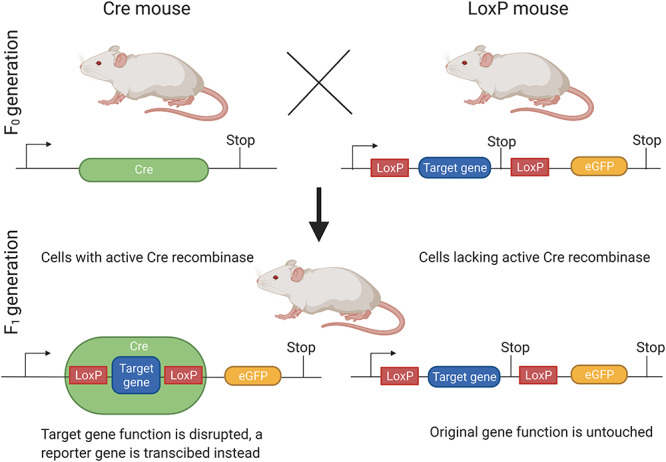
The Cre/LoxP system used to generate ARKO mice. *ARKO* androgen receptor knockout, *eGFP* enhanced green fluorescent protein

Global knockout of the AR results in inactivation of AR expression in all tissue. In humans, an inactivating mutation of the *Ar* gene leads to complete androgen insensitivity syndrome (CAIS) (formerly known as testicular feminization), which represents complete resistance to androgenic action, resulting from the formation of defective or inert ARs with inhibition of all subsequent AR-mediated signaling [7]. The first rodent model of CAIS was described in the testicular-feminized rat by Stanley-Gumbreck in the early 1960s [28, 29]. In the 1970s, Lyon identified a comparable mouse line with X-linked complete androgen insensitivity [28, 29]. Later studies demonstrated that CAIS with non-functional AR is caused by a natural single point deletion in the N-terminal domain of the *Ar* gene [30, 31]. As the AR is located on the X chromosome, female homozygous mice with an inactivated AR could not be produced by natural mating as the hemizygous male fathers with an inactive AR are sterile [32]. More recently, AR knockout (ARKO) female mice have been generated using the efficient Cre/LoxP system [33–36], which is a conditional gene-targeting method that can be used to generate global or cell-specific ARKO mice [37]. In our research group, we have established an in-frame *Ar* exon 3 deletion ARKO female model that is reported to have reduced litter sizers but exhibit normal follicle population of at least up to 16 weeks of age [35, 36]. The Cre/loxP system involves the target gene being flanked by loxP sites, thus becoming “floxed”, and subsequently cut by a bacterial Cre recombinase enzyme [38]. Using this technology, the knockout mice are produced by crossing transgenic mice that express the Cre recombinase enzyme with mice carrying a floxed exon in the target gene. The conditional gene knockout using the Cre/loxP strategy will involve selecting a specific promoter to drive Cre recombinase expression in the tissue of interest (Figure 3).

Global AR-knockout mice have been developed using the transgenic Cre lines with global Cre expression (i.e. Cmv-Cre) crossed with floxed AR mouse lines. Currently, five floxed AR mouse lines involving loxP sites flanking the AR gene at exon 1(AR^Ex1^) [39, 40], exon 2 (AR^Ex2^) [33, 41] and exon 3 [42] exist (Table 2). These floxed AR models have been crossed with different transgenic Cre lines to generate global ARKO models. In the cases of both AR^Ex1^KO and AR^Ex2^KO mice, which rely on the major loss of AR protein or deletion of the respective exons from the *Ar* gene, the introduction of a premature stop codon prevents the transcription to AR protein. In contrast, deletion of exon 3, which encodes for the second zinc finger in the DNA binding domain, only results in a minimally truncated, but inactive, AR with normal androgen-binding affinity, but which is unable to bind to DNA, consequentially disabling downstream gene transactivation [43, 44].

**Table 2:** Summary of five ARKO mouse models established

	**Floxed exon**	**Universal deletor Cre recombinase**	**Reference**
ARKO line 1	Exon 2	b-Actin	[33, 45]
ARKO line 2	Exon 2	PGK	[41]
ARKO line 3	Exon 1	CMV	[46]
ARKO line 4	Exon 1	Sycp1 and ella	[39]
ARKO line 5	Exon 3	CMV	[35, 42]

*ARKO* androgen receptor knockout

#### Cell-specific knockout mouse models

To study the role of androgens in specific cells and tissues, over 25 cell-specific knockout mice have been generated [24, 34]. To study the specific role of androgen actions mediated via the AR in skin and wound healing, keratinocytes, dermal fibroblasts and infiltrating inflammatory cell-specific AR-knockout mice have been established. Using cell-specific Cre-Lox models (Table 3), AR has been knocked out in myeloid cells (MARKO), keratinocytes and fibroblasts [23, 47–50]. These cell-specific models exhibit AR protein deficiency in target cells, with functional AR expressed normally throughout the rest of the body. However, the transgenic Cre lines can have limitations that need to be considered, such as the Cre activity not having 100% efficiency (mosaic knockout in target cells) or specificity (off-target knockouts). This may not be evaluated or reported in the studies.

### Effects of androgens on the cutaneous wound-healing process

**Figure 4.**
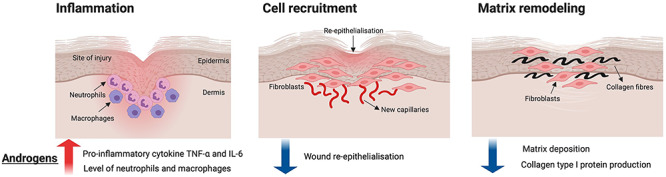
Summary of androgen functions in cutaneous wound healing. The red arrow indicates the increase of cytokine TNF-α and IL-6, as well as the neutrophils and macrophage populations. The blue arrows indicate the decrease of wound re-epithelialisation and matrix deposition. *TNF-α* tumor necrosis factor-α, *IL-6* interleukin-6

Cutaneous wound healing is a complex process with many factors, such as stress, infection and malnutrition, contributing to impaired wound healing [53]. Cutaneous wound healing is slower in men when compared to women [31, 32] and this gender-related distinction suggests that sex hormones may play an important role in wound healing. Further investigations on the specific role of androgens in the different stages of wound healing have since been performed in better-controlled experimental mouse models. Murine models established to study the role of androgens in wound healing include orchidectomy [18–21]; AR blockade with the AR antagonist flutamide [18, 49]; the 5α-reductase inhibitor MK-434 [20] and AR degradation enhancer ASC-J9 [23]; or global ARKO mice [23]. In these studies, androgens display an inhibitory role, delaying cutaneous wound healing in male mice (Figure 4)—an effect that is reversed by treatment with exogenous androgens in orchiectomized mice [13, 19, 49].

**Table 3:** Summary of cell-specific knockout mouse models used in skin studies

**Cell type**	**Promoter for Cre recombinase**	**Target**	**Functions**	**Study**
Keratinocyte knockout	Keratin 5	Keratinocyte in cutaneous wound healing	Modulating epidermal migration, inhibit re-epithelization	[23]
Fibroblast knockout	FSP1	Stromal fibroblast in prostate	Increased apoptosis, decreased epithelial proliferation and collagen composition	[51, 52]
	FSP1	Fibroblast in cutaneous wound healing	Modulating epidermal migration, promote re-epithelization	[23]
Myeloid cell knockout	Lysozyme M	Macrophage	Up-regulate IL-6 and TNF-α production, prolong inflammation	[23]

*FSP1* fibroblast-specific protein1, *TNF-α* tumor necrosis factor-α, *IL-6* interleukin-6

#### Androgens and inflammation

Interleukin-6 (IL-6) and tumor necrosis factor-α (TNF-α) are pro-inflammatory cytokines that are upregulated during the inflammation phase of wound healing. IL-6 increases keratinocyte proliferation and is chemoattractive to neutrophils, [54] while TNF-α inhibits wound re-epithelization. Transforming growth factor-β1 (TGF-β1) delays re-epithelization but stimulates wound contraction and matrix deposition. Androgens exhibit inflammatory properties by mediating the expression of various cytokines [55, 56], which has prompted research into the inflammatory tendencies of androgens in wound repair. Castration (absence of DHT and testosterone) reduces wound TNF-α production and macrophage infiltration in mice [18, 22, 54]. Similar results have been observed in castrated rats, which display reduced IL-6 and TNF-α expression and elevated TGF-β1 levels associated with decreased wound neutrophil influx [20, 22]. These results suggest that androgens prolong inflammation in wound healing by promoting the local expression of pro-inflammatory cytokines, such as TNF-α and IL-6, and wound infiltration of inflammatory cells. In a study by Gilliver *et al.* [22], rats treated with a 5α-reductase inhibitor (which prevents the conversion of testosterone to DHT), displayed faster wound repair, reduced IL-6 expression and decreased influx of inflammatory cells, but only minor effects on TNF-α and TGF-β1 expression in wounds. These findings suggest that IL-6 is predominantly regulated by DHT, whereas TNF-α and TGF-β1 are regulated by testosterone. These studies demonstrate that significantly reducing circulating potent androgens reduces inflammation by preventing the excessive infiltration of immune cells and the production of key pro-inflammatory cytokines, highlighting the role of androgens as natural inhibitors of wound repair [22].

Mice treated with topical flutamide, an AR antagonist, were reported to have shown significantly accelerated wound healing with reduced tissue expression of TNF-α [18], suggesting that blocking DHT production reduces local tissue inflammation and modulates wound repair. In a more recent study, conditional cell-specific knockout of AR in mice has been used to investigate the specific role of AR in the wound-healing process [23]. Global ARKO and MARKO mice also exhibited accelerated wound healing compared to wild-type controls [23], highlighting the role of AR in retarding wound repair in males. TNF-α production was reduced in the wounds of both knockout models, and the local restoration of TNF-α resulted in impaired wound healing in global ARKO mice. The production of IL-6 was also decreased in MARKO mice [23]. These findings suggest that AR, specifically expressed by myeloid cells, play a critical role in androgen action mediated local upregulation of TNF-α and IL-6. Taken together, androgens augment the inflammatory response by promoting cytokine production and the infiltration of inflammatory cells that contribute to prolonged wound repair.

#### Androgens in cell-recruitment phases of wound repair

The role of androgens in the cell-recruitment phase of wound healing has also been extensively studied. Gilliver and colleagues showed that 5α-reductase inhibition in rats resulted in faster wound re-epithelialization, suggesting that DHT hinders epithelial formation and contributes to impaired wound healing in male rats [48]. Enhanced re-epithelialization was also observed in global ARKO mice; however, there were no changes in cell proliferation reported [23]. These findings highlight the role of AR in suppressing wound re-epithelialization by modulating epidermal migration, rather than cell proliferation. Although AR knockout in fibroblasts accelerates re-epithelialization, AR-specific knockout in keratinocytes showed no effect on cell proliferation [23]. This suggests that AR in fibroblasts and keratinocytes inhibits and promotes re-epithelialization, respectively. Nonetheless, the effects of AR knockout in fibroblasts may overcome those effects of AR knockout in keratinocytes, resulting in an overall increase in re-epithelialization in global AR knockout. The role of AR in impaired wound healing was further confirmed by Toraldo *et al.* [36], who demonstrated that AR antagonism with topical application of flutamide accelerated re-epithelialization and enhanced wound repair in mice. As β-catenin delays healing [57] and hinders in vitro keratinocyte migration [58], Gilliver *et al.* investigated the role of β-catenin further. Their study showed that androgens impede re-epithelialization by positively regulating β-catenin, and that DHT deficiency in rats was associated with reduced β-catenin protein levels and advanced epidermal formation [48]. Taken together, androgens acting on AR delays re-epithelialization during the cutaneous wound-healing process, potentially by stimulating β-catenin production.

#### Androgens modify matrix remodeling

The matrix remodeling phase involves the secretion of collagens type I, III and IV and fibronectins and proteoglycans from epithelial cells [59]. Collagen type I is constantly organized to transform the provisional matrix into a mature extracellular matrix. Matrix remodeling is governed by the controlled cleavage of collagen type I through collagenases and gelatinases [60]. Wounds of castrated animals exhibit elevated levels of collagen type I [18, 61] and fibronectin [61], contributing to faster wound healing. These findings suggest that androgens delay matrix deposition during wound repair. In addition, the production and activity of collagenases, matrix metalloproteinase (MMP)-1 and MMP-13, and gelatinases, MMP-2 and MMP-9. was reduced in castrated rats and associated with increased collagen accumulation in the wound area [61]. These results demonstrate that androgens encourage the production, and thereby the proteolytic properties, of both collagenases and gelatinases, leading to increased collagen I degradation in wounds during the matrix remodeling phase. In global ARKO and MARKO mice, an increase in wound collagen content was apparent. However, cell-specific AR knockout in both fibroblasts and keratinocytes had no effect on the level of collagen accumulation [23]. This implicates cell-specific involvement of AR in attenuating collagen deposition during wound repair. Overall, androgenic actions via AR have a negative effect on matrix deposition, possibly by stimulating the synthesis and enzymatic activity of collagenases and gelatinases to degrade collagen type I proteins.

### Androgens in major burn injury wound healing

In contrast to cutaneous injury, major burn injury initiates both cutaneous local wound-healing processes as well as a systemic hypermetabolic response. Patients with a 20% total body surface area burn become hypermetabolic, experiencing a significant increase in resting energy expenditure, which is largely driven by elevated levels of circulating catecholamines, corticosteroids and pro-inflammatory cytokines following the burn injury. Furthermore, hypermetabolic burns patients can suffer from endocrine dysfunction, immune compromise, insulin resistance and whole-body catabolism [62, 63]. These clinical features are associated with delayed recovery, prolonged hospital admission and increased morbidity and mortality [62, 63]. Currently available treatments to ameliorate hypermetabolism in major burn patients include early excision and closure of the wound, nutritional support or pharmacologic modalities, such as androgens and other anabolic hormones [64]. Clinical studies have reported that testosterone and the synthetic androgen oxandrolone can enhance recovery from burn injury [65]. Androgen-treated burns patients have been reported to maintain more lean body mass and have improved body composition and hepatic protein synthesis during the acute postburn phase [66, 67]. Although there are reports of improved patient outcomes, mainly in children with major burns, the role of androgens in major burn injury wound healing is not clear. Androgen treatments have not been generally adopted due to the lack of clarity of the mechanism of ameliorating the hypermetabolic response, as well as the risks of hepatotoxicity from a 17 α-alkylated androgen and unwanted virilization of women and children.

**Table 4:** Clinical studies of oxandrolone use in patients with major burn injury

**Patient information**	**Dose of oxandrolone treatment**	**Duration of oxandrolone treatment**	**Primary outcomes**	**Study**
**Acute phase post-burn**				
Oxandrolone (n = 7) Placebo (n = 7) <18 years >20% TBSA	0.1 mg/kg oral twice daily	1 week	Increased muscle protein net balance, protein synthesis efficiency and muscle protein breakdown	[1]
Oxandrolone (n = 46) Placebo (n = 35) >18 years 20-60% TBSA	10 mg oral or via enteral feeding tube every 12 hours	Beginning 5 days after injury, stopped halfway due to significant difference found between group	Significant reduction of length of hospital stay	[81]
Oxandrolone (n = 11) Placebo (n = 9)	20 mg/day	Beginning between days 2 and 3 post-burn, average 33 ± 9 days until transfer to rehabilitation	Decreased weight loss and net protein loss Increased donor site wound healing	[76]
Oxandrolone (n = 16) Placebo (n = 24)	20 mg/day	Beginning between days 7–10 post-burn monitoring until transfer to rehabilitation	Decreased weight loss and net protein loss Increased donor site wound healing	[70]
Oxandrolone (n = 59) Placebo (n = 58)	Not available	Administration within 7 days after admission with a duration of at least 7 days	Increased survival rate	[83]
Oxandrolone (n = 7) Placebo (n = 7) <18 years	0.1 mg/kg twice daily	5 days	Increased protein synthesis, altered gene expression but no effect on protein breakdown	[84]
Oxandrolone (n = 45) Placebo (n = 190) <18 years	0.1 mg/kg twice daily	30 days	Significantly reduced length of intensive care unit stay Increased LBM and muscle strength	[71]
**Recovery phase post-burn**				
Oxandrolone (n = 30) Placebo (n = 31) ≤18 years ≥40% TBSA	0.1 mg/kg twice daily	12 months post-burn	Increased lean body mass, bone mineral content and muscle strength	[85]
Oxandrolone (n = 35) Placebo (n = 84) 0–18 years ≥30% TBSA	0.1 mg/kg Oral Twice daily	12.1–25.2 months post-burn	Increased whole-body bone mineral content, lumbar spine bone mineral content and density	[86]
Oxandrolone (n = 12) Placebo (n = 10) ≤18 years ≥40%TBSA	0.1 mg/kg twice daily	6 months post-burn	Increased net deposition of leg muscle protein but no effect on whole-body protein breakdown	[80]
Oxandrolone (n = 14) Placebo (n = 18) <18 years	0.1 mg/kg twice daily Oral or via feeding tube	From 7 days after acute admission for the duration of hospitalization	Increased body weight, fat-free mass after treatment	[82]
Oxandrolone (n = 10) Placebo (n = 11) TBSA ≥40% <18 years	0.1 mg/kg twice daily	12 months post-burn	Increased constitutive protein level and decreased acute phase protein	[65]
Oxandrolone (n = 42) Placebo (n = 42)	0.1 mg/kg twice daily	12 months post-burn	Increased lean body mass and bone mineral content Clitoromegaly with oxandrolone in 2 children, leading to oxandrolone discontinuation	[77]

*TBSA* total body surface area burned

#### Testosterone treatment

Serum testosterone levels are decreased significantly in burn patients in an intensive care unit during the first week post injury [68]. Whether decreased serum testosterone contributes to the burn-induced catabolic stress state is not known. Testosterone administration has been reported to have positive effects on patients with burn injuries and mainly manifests through better preservation of muscle [69]. Testosterone administration has been shown to reduce the rate of protein breakdown and increase the net protein balance in muscle during the acute burn injury phase, mitigating protein catabolism of the systemic hypermetabolic response to major burn injury. Protein synthesis did not change with testosterone treatment and was attributed to the limited availability of precursor amino acids [69]. Testosterone treatment has therefore been proposed to provide an antagonistic balance to the hypermetabolic serving to reduce protein breakdown without necessarily increasing protein synthesis. Current studies investigating the effects of testosterone treatment following burn injury has been limited by small sample sizes, lack of placebo control and a limited range of important clinical outcomes being assessed (e.g. wound healing, length of hospital stay). Furthermore, many of these studies have only been conducted in male burn patients.

**Figure 5.**
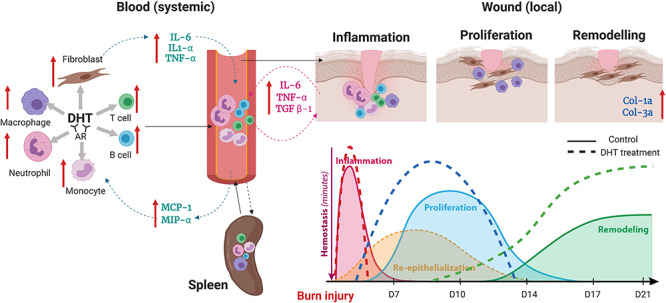
Illustration of DHT enhance major burn injury wound healing via accelerating inflammationturnover, resulting in a fast resolution of inflammation phase followed by early proliferation and remodelling. The red arrows indicate the increase in immune and fibroblast cell population and inflammatory markers concentration after systemic administration of DHT in mice model. *TGF-β* transforming growth factor-β, *MCP-1* monocyte chemoattractant protein-1, *MIP-α* acrophage inflammatory protein α, *TNF-α* tumor necrosis factor-α, *IL-6* interleukin-6, *DHT* dihydrotestosterone, *AR* androgen receptor

#### Oxandrolone in treating major burn injuries

Major burn injury causes significant depletion in lean body mass, resulting in complications such as infection, failure to thrive and endocrine, immune and nutritional deficiencies due to increased metabolic demands and inefficient energy utilization. Previous studies have reported that the administration of oxandrolone had no significant effect on resting expenditure rate or basal metabolic rate when measured by indirect calorimetry, suggesting that oxandrolone does not reduce the burn-induced hypermetabolic state [1, 70–72]. However, some clinical studies found that combining oxandrolone with exercise consistently improved the gross parameters of lean body mass, total body mass, bone mineral composition, strength and reduced length of hospital stay in patients with major burn injuries (Table 4) [1, 70–82]. A majority of these studies noted some benefit to net muscle deposition or fractional synthesis rate in oxandrolone-treated patients compared to controls via either an increase in protein synthesis or a decrease in protein breakdown (Table 4) [1, 65, 69–71, 76, 77, 80, 82–87]. Increased protein synthesis in patients treated with oxandrolone may be associated with the upregulation of genes governing transcription factors, growth factors and muscle-associated proteins, including myosin, light chain and calmodulin, while also downregulating phosphatase I inhibitor [82, 88].

Oxandrolone also stimulates an upregulation of supplementary systemic anabolic hormones, including insulin-like growth factor-1 and thyroid hormones, all of which promote protein synthesis, increasing lean body mass [72, 78, 79]. Moreover, oxandrolone has been demonstrated to increase inflammatory markers and hepatic acute phase proteins, such as ferritin, haptoglobin, C-reactive protein and α2-macroglobin, returning the levels to normal at a faster rate compared to controls [71]. It has also been claimed that oxandrolone may inhibit glucocorticoid action via ARs, thus limiting systemic catabolism and proteolysis [71]. Whether the use of oxandrolone in clinical studies will compromise adrenal response to severe injury remain to be investigated. However, the effects of oxandrolone, and that of safer, alternative androgens, on local wound-healing processes and systemic induced hypermetabolism have not been demonstrated and therefore require further investigation, particularly to better understand the role of androgen action in major burn injury recovery. Proponents of oxandrolone claim it has decreased side effects compared to testosterone but few studies with testosterone are reported and none have compared it to oxandrolone [70, 74, 77, 81, 85, 89]. The major concerns for oxandrolone are unwanted virilization of women and children and hepatoxicity, both of which remain to be clarified [65, 77, 79].

#### DHT in treating major burn injuries

DHT is the most potent natural androgen, with higher affinity and greater molar potency in the transactivation of the AR. DHT lacks the hepatotoxicity of 17 α-alkylated synthetic androgens, highlighting its overlooked potential as an important therapeutic treatment option. Aside from a few studies showing the effect of DHT treatment on increasing mouse skeletal muscle size and strength, research on the application of DHT in burn injury wound healing are limited [90, 91]. In our recent study, the effect of DHT treatment in major burn injury healing was examined in a burn injury mouse model [92, 93]. Our data showed that DHT has a positive impact on both local wound healing and metabolic catabolic responses, which differs from results reported after cutaneous injury. Mice that received DHT implantations had a faster healing rate, particularly in the early stages of the healing process. While those cirulating immune cell then infiltrate into the wound site and help with the resolution of inflammation. The increase in monocyte chemoattractant protein-1 (MCP-1) level at the wound area and systemically recruits more monocytes to the blood circulation and the wound site. These monocytes were found to differentiate to macrophages, which are involved in removing bacteria, preventing infection and contributing to collagen disposition once the inflammation is resolved [93] (Figure 5). Therefore, as a safer, non-hepatoxic androgen, DHT was approved for the first time to induce acceleration of the inflammatory turnover both locally and systemically as the key in promoting major burn injury wound healing without any adverse effects.

## Conclusions

This review summarizes the current understanding of the role of androgens in both cutaneous and major burn injury wound healing. The inhibitory role of androgens in cutaneous wound healing has been well-studied with androgens modifying the inflammation and delaying the proliferation phase. In major burn injury, a systemic hypermetabolic state develops and the administration of testosterone or oxandrolone have been clinically reported to improve maintenance of body weight, increase muscle protein metabolism and shorten hospital stay. However, at present, the role of safe androgens in major burn injury wound healing is poorly understood and further investigation into the therapeutic potential of androgens for major burn injury patients is warranted.
